# Comparative use of exercise tolerance testing, SPECT and CMR, alone and in combination, for the diagnosis of coronary heart disease in the CE-MARC study

**DOI:** 10.1186/1532-429X-15-S1-O10

**Published:** 2013-01-30

**Authors:** John P Greenwood, Neil Maredia, Julia Brown, John Younger, Colin Everett, Jane Nixon, Catherine J Dickinson, Sven Plein

**Affiliations:** 1 LIGHT Institute, University of Leeds, Leeds, UK; 2 Clinical Trials Research Unit, University of Leeds, Leeds, UK; 3 Department of Nuclear Cardiology, Leeds Teaching Hospitals, Leeds, UK

## Background

Whilst exercise tolerance testing (ETT) has been a corner stone investigation for the diagnosis of patients with suspected angina, increasingly imaging techniques have gained prominence. We aimed to determine the diagnostic accuracy of ETT in the CE-MARC study population compared to single photon emission computed tomography (SPECT) and cardiovascular magnetic resonance (CMR) and to examine the clinical utility of performing CMR or SPECT after an inconclusive ETT result.

## Methods

CE-MARC was the largest prospective real-world evaluation of CMR, SPECT and ETT, in 752 patients with suspected angina. Results for CMR and SPECT have been reported. For this analysis, results of the ETT were analysed and compared with CMR and SPECT as well as combinations of tests.

## Results

580 patients had ETT and angiography (disease prevalence 39%). The sensitivity, specificity, positive and negative predictive values (95%CI) of ETT were 68.3 (61.9, 74.0), 72.5 (67.6, 76.9), 61.0 (54.8, 66.8), 78.4 (73.7, 82.5). All four study tests (ETT, CMR, SPECT and coronary angiography) were undertaken in 503 patients, in which SPECT or CMR were used to adjudicate on the inconclusive ETT tests. Combined ETT and CMR had significantly superior sensitivity and negative predictive value compared to combined ETT and SPECT (P=0.0266, P=0.0114 respectively) but the specificities and PPV's were similar. Combined ETT and SPECT compared to SPECT alone resulted in improved sensitivity (86.3% vs. 65.5%) and negative predictive value (79.0% vs. 87.9%) but at a cost of reduced specificity and positive predictive values (83.7% vs. 64.1%; 72.1% vs. 60.7%). Overall, the combined ETT and CMR strategy did not outperform CMR alone.

## Conclusions

CMR provides more accurate diagnostic information than ETT, SPECT or a combination of the two, in patients with suspected stable angina.

## Funding

CE-MARC was funded by the British Heart Foundation (BHF). SP is funded by a BHF fellowship.

**Figure 1:**
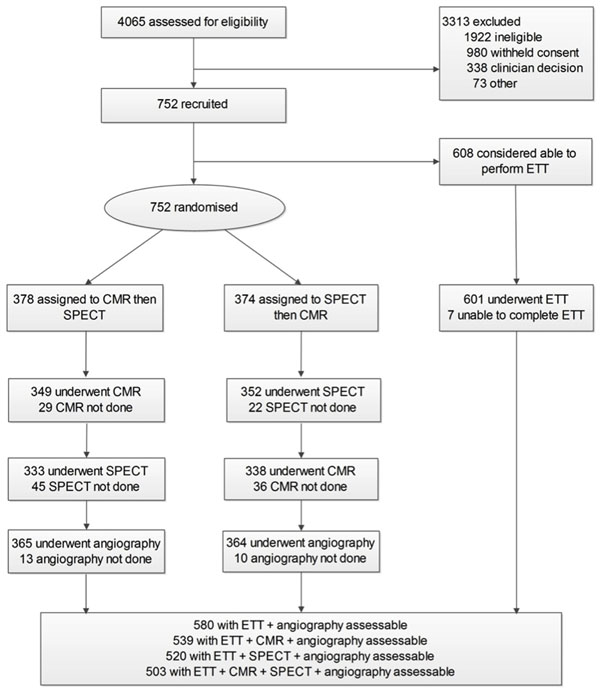
Patient flow diagram.

